# 2535. Physiological-based pharmacokinetic analysis of drug–drug interactions between isavuconazole and vincristine in pediatric subjects

**DOI:** 10.1093/ofid/ofad500.2152

**Published:** 2023-11-27

**Authors:** Mary P Choules, Yukio Otsuka, Laura Kovanda, Peter Bonate, Shamim Sinnar, Amit Desai

**Affiliations:** Astellas Pharma Global Development Inc., Northbrook, Illinois, USA, Northbrook, Illinois; Astellas Pharma Inc., Tokyo, Japan, Tokyo, Tokyo, Japan; Astellas Pharma Global Development Inc., Northbrook, Illinois, USA, Northbrook, Illinois; Astellas Pharma Global Development Inc., Northbrook, Illinois, USA, Northbrook, Illinois; Astellas Pharma Global Development Inc., Northbrook, Illinois, USA, Northbrook, Illinois; Astellas Pharma Global Development, Northbrook, IL, Northbrook, Illinois

## Abstract

**Background:**

Invasive fungal diseases (IFD), including invasive aspergillosis (IA) and invasive mucormycosis (IM), occur primarily in immunocompromised and/or hospitalized pediatric patients and are associated with morbidity and mortality. Pediatric patients being treated for leukemia with vincristine frequently experience secondary fungal infections, requiring treatment. Isavuconazole (ISAV) is approved for the treatment of IA/IM in adults; but there is limited data for its use in pediatric patients. Isavuconazonium sulfate is the prodrug of the active triazole ISAV, an antifungal agent that inhibits sterol 14α-demethylase. Isavuconazonium is rapidly converted by plasma esterases to ISAV, which is subsequently metabolized by the liver through CYP3A. ISAV was found in clinical drug–drug interaction (DDI) studies to be a moderate inhibitor of CYP3A, an inducer of CYP2B6, and to have inhibitory potential for P-gp and OCT-2 transporters. Because vincristine is eliminated hepatically via CYP3A and biliary excretion through P-gp, there is a potential for interaction with triazoles. Therefore, a physiological-based pharmacokinetic (PBPK) analysis was conducted to assess the potential for interaction between ISAV and vincristine.

**Methods:**

PBPK models were built for ISAV and vincristine in a pediatric cancer population using Simcyp^®^ simulator software with in vitro and in vivo data. The final verified models were used to predict change in vincristine exposure following coadministration with ISAV.

**Results:**

The predicted geometric mean (GM) area under the curve (AUC) ratio of vincristine co-administered with ISAV ranged from 1.54-fold to 1.69-fold depending on the dosing scheme used for the prediction as detailed in Table 1. Coadministration of vincristine when ISAV was at steady state (14 days) did not result in a significant difference in the magnitude of DDI from other dosing schemes. No effect was predicted on the GM C_max_ ratio of vincristine regardless of the ISAV dosing scheme.

**Figure:**
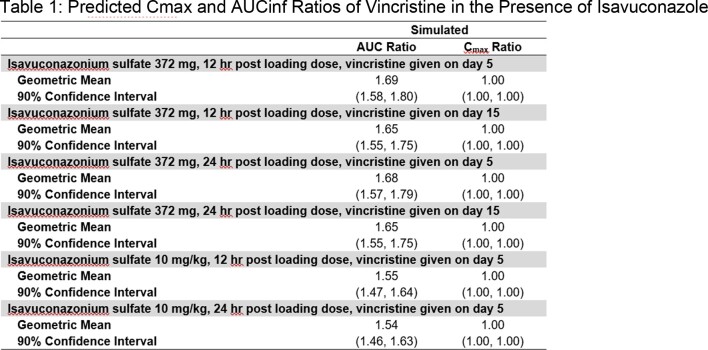

**Conclusion:**

ISAV is predicted to be a weak inhibitor of vincristine elimination in pediatric cancer participants, and DDI modelling predicts a slight increase in vincristine exposure when vincristine is co-administered with ISAV.

**Disclosures:**

**Mary P. Choules, PharmD, PhD**, Astellas Pharma Global Development, Inc.: Astellas Employee **Yukio Otsuka, MSc**, Astellas Pharma Global Development Inc.: Astellas Employee|Astellas Pharma Global Development Inc.: Stocks/Bonds **Laura Kovanda, PhD**, Astellas Pharma Global Development Inc.: Astellas Employee **Peter Bonate, PhD**, Astellas Pharma Global Development, Inc.: Astellas Employee **Shamim Sinnar, MD, PhD**, Astellas Pharma Global Development, Inc.: Astellas Employee **Amit Desai, PhD**, Astellas Pharma Global Development, Inc.: Astellas Employee

